# Integration of Autonomous Wireless Sensor Networks in Academic School Gardens

**DOI:** 10.3390/s18113621

**Published:** 2018-10-25

**Authors:** Peio Lopez-Iturri, Mikel Celaya-Echarri, Leyre Azpilicueta, Erik Aguirre, José J. Astrain, Jesús Villadangos, Francisco Falcone

**Affiliations:** 1 Department of Electric, Electronic and Communication Engineering, Public University of Navarre, 31006 Pamplona, Spain; peio.lopez@unavarra.es (P.L.-I.); erik.aguirre@unavarra.es (E.A.); francisco.falcone@unavarra.es (F.F.); 2 Institute for Smart Cities, Public University of Navarre, 31006 Pamplona, Spain; josej.astrain@unavarra.es (J.J.A.); jesusv@unavarra.es (J.V.); 3 School of Engineering and Sciences, Tecnologico de Monterrey, 64849 Monterrey, NL, Mexico; mikelcelaya@gmail.com; 4 Department of Mathematical Engineering and Computer Science, Public University of Navarre, 31006 Pamplona, Spain

**Keywords:** school garden, wireless sensor networks, energy harvesting, smart agriculture, EnOcean, MySchoolGardenApp

## Abstract

In this work, the combination of capabilities provided by Wireless Sensor Networks (WSN) with parameter observation in a school garden is employed in order to provide an environment for school garden integration as a complementary educational activity in primary schools. Wireless transceivers with energy harvesting capabilities are employed in order to provide autonomous system operation, combined with an ad-hoc implemented application called MySchoolGardenApp, based on a modular software architecture. The system enables direct parameter observation, data analysis and processing capabilities, which can be employed by students in a cloud based platform. Providing remote data access allows the adaptation of content to specific classroom/homework needs. The proposed monitoring WSN has been deployed in an orchard located in the schoolyard of a primary school, which has been built with EnOcean’s energy harvesting modules, providing an optimized node device as well network layout. For the assessment of the wireless link quality and the deployment of the modules, especially the central module which needs to receive directly the signals of all the sensor modules, simulation results obtained by an in-house developed 3D Ray Launching deterministic method have been used, providing coverage/capacity estimations applicable to the specific school environment case. Preliminary trials with MySchoolGardenApp have been performed, showing the feasibility of the proposed platform as an educational resource in schools, with application in specific natural science course content, development of technological skills and the extension of monitoring capabilities to new context-aware applications.

## 1. Introduction

Wireless Sensor Networks are being actively adopted as enablers for context aware communication capabilities within multiple scenarios, such as Smart Cities and Smart Regions [1,2]. Within Smart City/Region concept, developments in the management of healthcare systems, water resources and waste, energy and transportation systems have been published in recent years [3,4,5,6,7]. Among these, agricultural management is gaining relevance since it concerns a fundamental aspect of human survival: feeding. As in the other mentioned cases, information and communication technologies have also been adopted in order to improve multiple aspects of agriculture [8]. In this context, WSNs play a key role, given to the fact that they constitute inherent distributed systems, in which current platforms allow the inclusion of multiple analogue/digital input/output ports. Furthermore, the use of wireless communication systems enables ubiquity as well as ease of deployment. Multiple challenges must also be handled, such as compact form factors, reduced energy consumption, interference handling and variable node density allocation. In this sense, HetNet solutions as well as incumbent 5G systems provide user access/interference control mechanisms, which rely on radio propagation characteristics in site-specific fashion, such as Self Optimizing Networks, Cloud RAN or Cooperative MAC schemes [9]. Adequate network operation and design require wireless channel analysis and optimization in order to minimize interference, energy consumption and enhance overall quality of service. This is of particular interest in the case of wireless sensor networks, given inherent restrictions in their operating conditions, as well as in the potentially large number of nodes present in the network.

All these advancements lead to the so-called Precision Agriculture and Smart Agriculture, where the information gathered by sensors (such as environmental parameters [10] or chemical component and soil water detection [11]) provide support to decision systems [12], facilitating the adoption of measures in order to optimize resources such as water or fertilizers [13,14,15,16], control and manage plant growth [17], as well as to detect, prevent and treat diseases [18,19]. One of the main advantages that this kind of systems can provide is the possibility to control and act from a location far away from the crops. This is feasible by means of IoT systems, which send to a Cloud (through a gateway) the information gathered by the WSN deployed on the crops [20]. Thus, the information can be stored and managed from any device connected to the Internet. In fact, there are a few works which present Cloud-based solutions for agricultural environment applications [21], a FIWARE-Based IoT platform for Precision Agriculture [22] and for hydroponic precision farming [23]. At this point, it is worth noting that commercial Smart Agriculture solutions are already in the market [24].

Regarding the communication between these devices, in most of the cases wireless technologies to deploy WSNs and IoT-based systems for Precision Agriculture and Smart Agriculture are used [25], such as Bluetooth [26] or GSM and Infrared communications [27]. But due to its ideal characteristics to deploy WSNs (low power consumption, low cost and high number of devices allowed per network), ZigBee is the most employed wireless technology [28,29,30,31,32,33], where transceivers are mounted and controlled by Arduino and Raspberry Pi boards in most cases. Even for precision agriculture based on mobile UAVs (Unmanned Aerial Vehicles) communicating with ground sensors, ZigBee has been used [34].

In this context, this work presents an educational application based on the combination of the capabilities provided by WSNs with parameter observation in a school garden in order to enhance the outcomes within the learning process of students in primary school. The novelty of the proposed system lies in two aspects of our work: On the one hand, since clean energy consumption for Smart Agriculture is gaining importance [35], Energy Harvesting (EH)-based EnOcean sensors have been used instead of the mostly used Raspberry Pi and Arduino-based devices. The main advantages of the commercial EnOcean devices are the much-reduced size of the motes and the EH system they have embedded. The drawback comparing to the Raspberry Pi and Arduino-based solutions present in the literature could be, for some applications, that the wireless network topology is limited to star topology type but in the presented application it is not a drawback due to the fact that all the motes are at a similar distance from the central node. On the other hand, the educational application itself is the second novel aspect of the presented study, since no other similar applications have been reported in the literature. Specifically, in this work wireless EnOcean transceivers with energy harvesting capabilities are employed in order to provide autonomous system operation, combined with an ad-hoc implemented application called MySchoolGardenApp. Information is retrieved in a cloud enable environment, providing remote data access and off-line processing capabilities, in order to adapt content to specific classroom needs. MySchoolGardenApp follows the trend marked by multiple initiatives within the educational community in which development environments such as Arduino/Genuino or Raspberry Pi are being employed in order to enhance learning outcomes in multiple disciplines, with a clear focus on Science, Technology, Engineering and Mathematics (STEM) [36,37,38].

The paper is organized as follows: Section 2 presents the scenario where the experiments have been done, the 3D Ray Launching simulation technique that has been used for the radio planning and the employed EnOcean devices for the creation of the WSN. Section 3 focuses on the radio planning simulation results and their analysis. In Section 4 the developed MySchoolGardenApp is presented and finally, in Section 5 the conclusions of the obtained results are commented.

## 2. Materials and Methods

**Figure 1:**
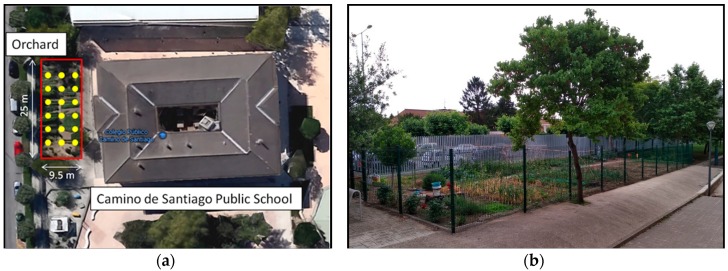
(**a**) ‘Camino de Santiago’ public school’s upper view; (**b**) A picture of the school’s orchard.

The experiments have been carried out in the orchard of the ‘Camino de Santiago’ primary school, located near the city of Pamplona. The orchard has an educational role, as the students learn how to grow different kind of vegetables such as cucumbers, pumpkins, onions, garlic, tomatoes, beans, zucchini, corn and so forth. The orchard is 25 m long and 9.5 m width and it is located within the school yard, near the school building, as can be seen in Figure 1a, where the orchard is delimited by a red rectangle. The 18 yellow dots within the red rectangle that can be seen in the figure represent the positions where the sensor devices of the proposed WSN have been placed. Figure 1b shows a real picture of the orchard under study.

**Figure 2:**
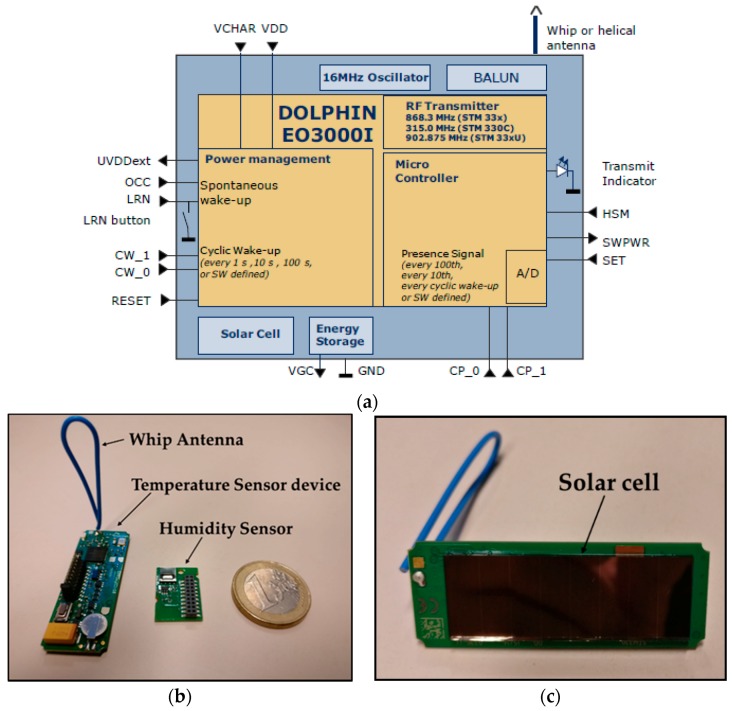
(**a**) Simplified device block diagram (extracted from STM 330 user manual). (**b**) EnOcean’s STM 330 module and the includable humidity sensor. (**c**) Detail of the STM 330 module’s solar cell.

The proposed WSN for monitoring the orchard has been built based on EnOcean’s energy harvesting modules. Specifically, the STM 330 modules have been employed, which employ the ISO/IEC 14543-3-1X proprietary standard for wireless communications. The STM 330 module makes use of the European operating frequency (868 MHz), receiver sensitivity value of −96 dBm@125 kbps and expected current consumption values ranging from 0.6 µA to 130 µA depending on charging and luminosity conditions. These wireless modules have integrated a 16 MHz 8051 CPU with 32 kB FLASH and 2 kB SRAM. They also have incorporated a temperature sensor (range 0 to 40 °C) and provide the possibility of equipping them with a humidity sensor, which has been used in this study as it provides interesting information for the purpose of the presented application. The humidity sensor is HSM 100 module, which has a measurement range from 0% to 100% with a resolution of 0.4% and a typical accuracy of ±5% (for values between 30–70%). In Figure 2a a simplified device block diagram is presented. Figure 2b shows a picture of a STM 330 module alongside the optional humidity sensor. In the same way, Figure 2c shows a picture of the opposite side of the same module, where the solar cell used for the energy harvesting can be seen. The power supplied by the solar cell is managed by an energy management circuit to bridge periods of darkness. Specifically, if the energy storage is fully charged, the operation time in darkness is typically 4 days (which could vary depending on the temperature), when the information transmissions are made every 1000 s. The module also provides user configurable cyclic wake-up functionality. After wake-up, a radio message is transmitted in case of a significant change of measured data is detected. Besides the reduced size of the modules, which provides an easy-to-deploy feature, it is very important to note that the energy harvesting technology of the devices (they are self-powered by the small solar cell) avoids the maintenance task of replacing batteries that common WSNs usually need. This is a very important feature of the EnOcean devices and it is worth noting that from the literature, there are very few solutions where self-powered devices are employed [13,33], the rest need to be powered externally as the use development boards such as Arduino or Raspberry Pi.

**Figure 3:**
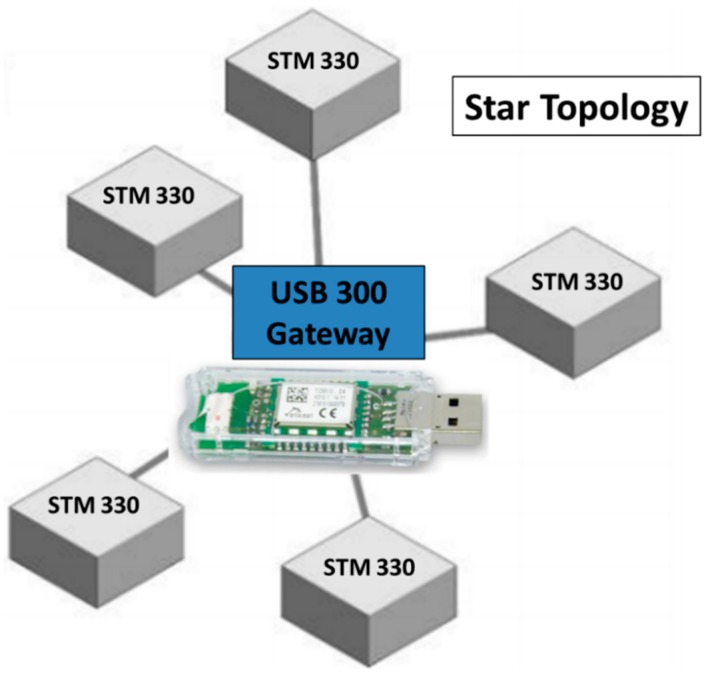
Schematic description of the star topology of the network with the EnOcean’s USB 300 Gateway central module.

Regarding the radio characteristics of the STM 330 modules, they operate at 868.3 MHz, which can provide longer distances than common devices operating at 2.4 GHz (such as ZigBee and Bluetooth) due to lower radio propagation losses. They provide a low data rate of 125 kbps (in comparison, ZigBee at 2.4 GHz transmits 256 kbps), which is enough in order to transmit the required information. The transmitted power level is between 5 and 8 dBm. The available network topology is much more restricted than other wireless technologies in terms of packets routing, as the only possible topology is the star topology (although the coverage of the network can be extended programming a device as a repeater), which means that each of the deployed STM 330 modules communicates only with a central module, usually connected to a PC or laptop via USB. Anyway, for the presented application, the benefits that provide these modules (size-deployment ease and energy harvesting system) make the EnOcean devices more interesting than those based on Arduino and Raspberry boards shown in the literature. Figure 3 shows a schematic description of the star topology of the network with the USB Gateway central module, which in this study case will be connected to a PC inside the school building. The Gateway records the temperature, humidity and RSSI value sent by each of the deployed STM 330 modules. The employed version of the Gateway has an internal chip antenna.

Before the implantation of the proposed system and the EnOcean-based WSN, a radio planning study has been performed in order to obtain data of the coverage of the WSN. This is particularly important in this case since the employed wireless devices have to be directly connected to the USB Gateway placed inside the building due to the restrictions of the star topology, that is, there are no routing elements in the network. For this task, an in-house developed simulation tool, called 3D Ray Launching, has been used. It is a deterministic method as it is based on the resolution of Maxwell’s equations but comparing to other deterministic methods it provides a good trade-off between precision and required computational time since it is simplified by ray launching and ray tracing techniques, based on geometrical optics. For the present study, the 3D Ray Launching technique will provide the RF power distribution for the whole 3D simulated scenario. This simulation tool has been broadly used and validated in both indoor and outdoor large scenarios [39,40]. It has also been tested satisfactorily for smart viticultural management [41].

## 3. Results

**Figure 4:**
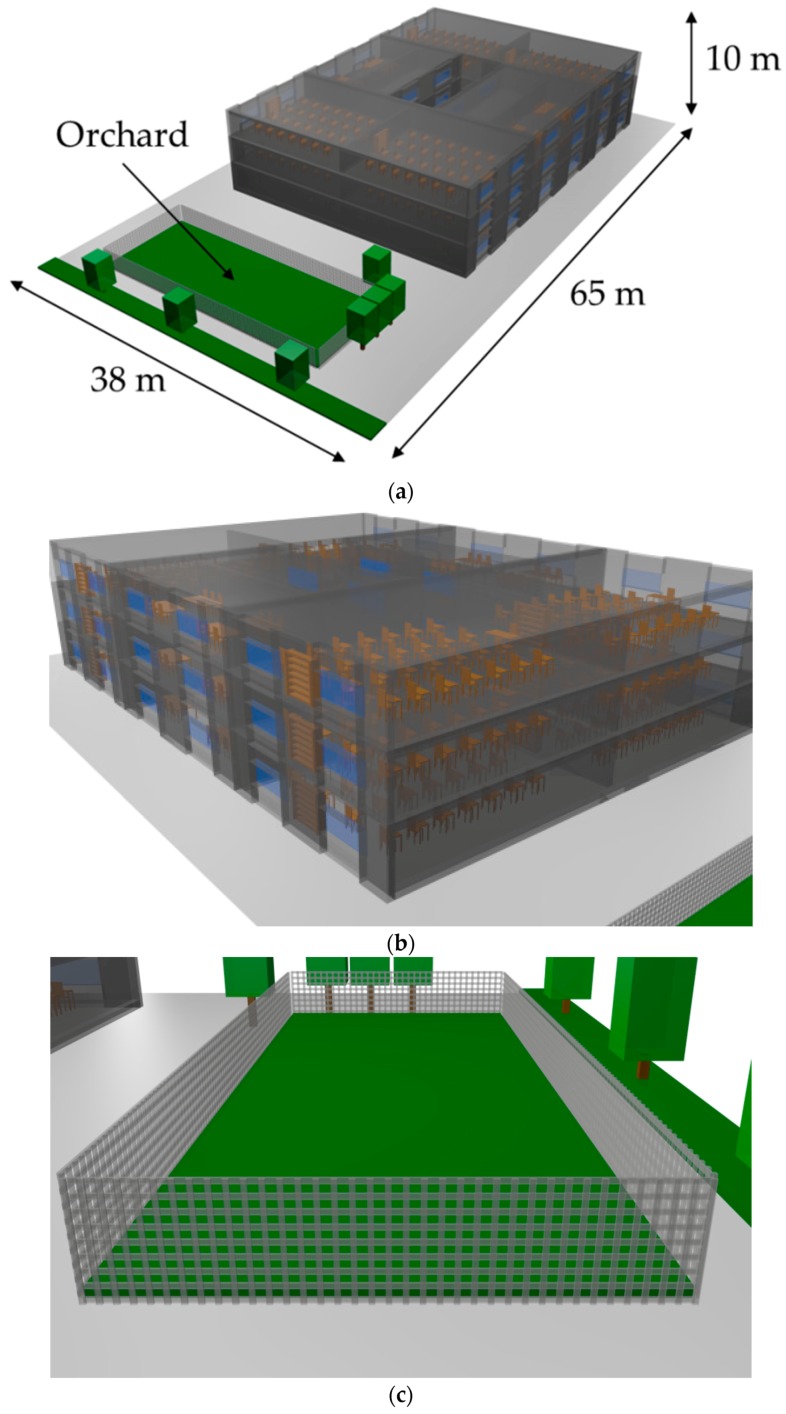
(**a**) Created scenario for the 3D Ray Launching simulations; (**b**) Detail of the building; (**c**) Detail of the metallic fence of the orchard.

As previously mentioned, a radio planning study has been performed in order to obtain information about the feasibility of the proposed EnOcean-based WSN for the monitoring of the school orchard. The scenario under analysis has been presented in Figure 1 and the created scenario for the simulations by means of the 3D Ray Launching tool is presented in Figure 4a. The simulated scenario is composed by the ‘Camino de Santiago’ public school building and the orchard which is part of its facilities. They are distributed in a 2470 m^2^ area scenario, where the orchard occupies 237.5 m^2^. The building has dimensions of 40 m long, 28 m width and 9 m height. It is worth noting that important elements in terms of its effect on radio propagation have been taken into account such as the interior of the school building, which has been filled approximately like the real building (see Figure 4b) and the metallic fence that surrounds the orchard (see Figure 4c). The material properties (dielectric constant and conductivity) of all the elements within the scenario, including organic materials for trees and orchard have also been considered.

**Figure 5:**
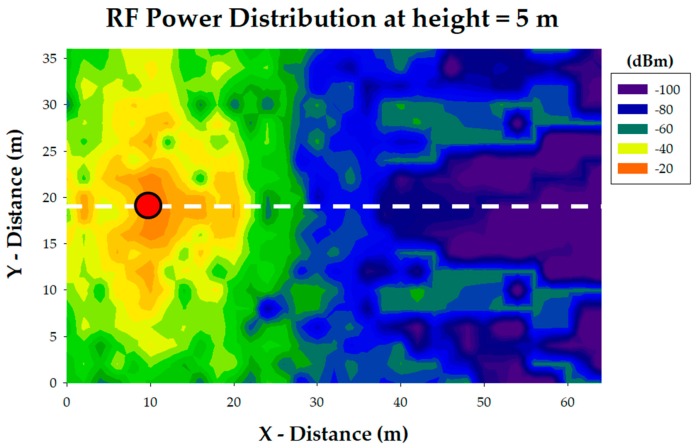
Estimated RF power distribution plane at height = 5 m for a wireless sensor on the orchard (represented by a red dot).

**Figure 6:**
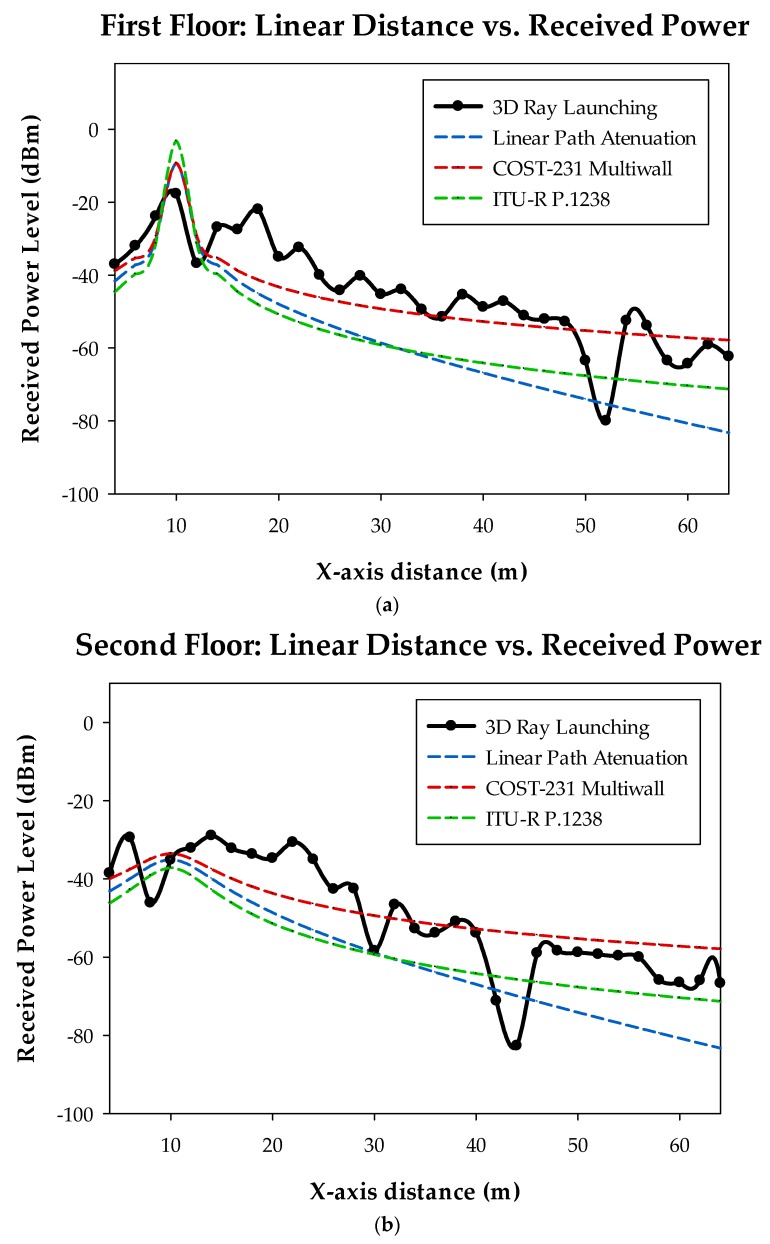

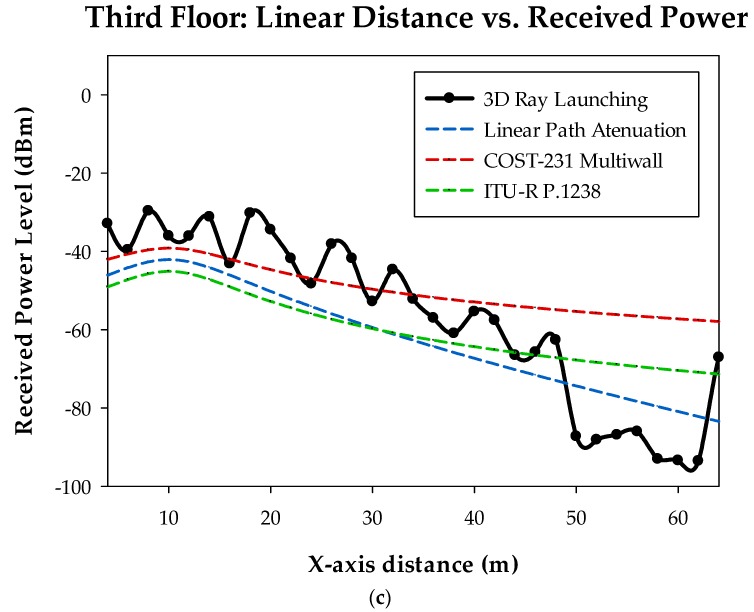
Linear path RF power distribution, corresponding to the white dashed line of Figure 5. (**a**) First floor height; (**b**) Second floor height; (**c**) Third floor height.

The main results provided by the 3D Ray Launching simulation tool are the RF power distribution planes. A transmitter is located within the created scenario and parameters such as transmission power level and antenna type are defined. Then, results for the whole volume of the scenario are obtained for each of the deployed wireless transmitters. As an illustrative example, Figure 5 shows the estimated values for a bi-dimensional plane at 5 m height for the simulation of one of the wireless sensors deployed on the orchard (represented by a red dot). The typical RF power distribution due to multipath propagation can be seen in the figure, which is caused by the morphology and topology of the considered scenario. Instead of the required process for 3D Ray Launching simulations, a faster methodology to obtain the received RF power at one point of the scenario has been also used, by means of empirical radio propagation models such as COST-231. These methods are based on measurement campaigns and provide very fast estimations. However, they lack precision since they do not take into account the multipath propagation which is specific for each scenario due to the elements within them such as furniture, columns, fences and so forth. In order to see clearer the difference between empirical methods and the 3D Ray Launching method, three linear path RF power distributions are shown in Figure 6. They correspond to the linear path marked by a white dashed line in Figure 5 and the presented heights (1.2 m, 4.25 m and 7.25 m) are equivalent to the tables’ heights of the first, second and third floor of the building, that is, the potential locations of the EnOcean gateway. As can be seen in all the graphs of Figure 6, the estimations provided by the 3D Ray Launching follow the tendencies of the other radio propagation models but they reflect the typical rapid variations generated by the multipath propagation, giving more precise results for an optimized radio planning.

**Figure 7:**
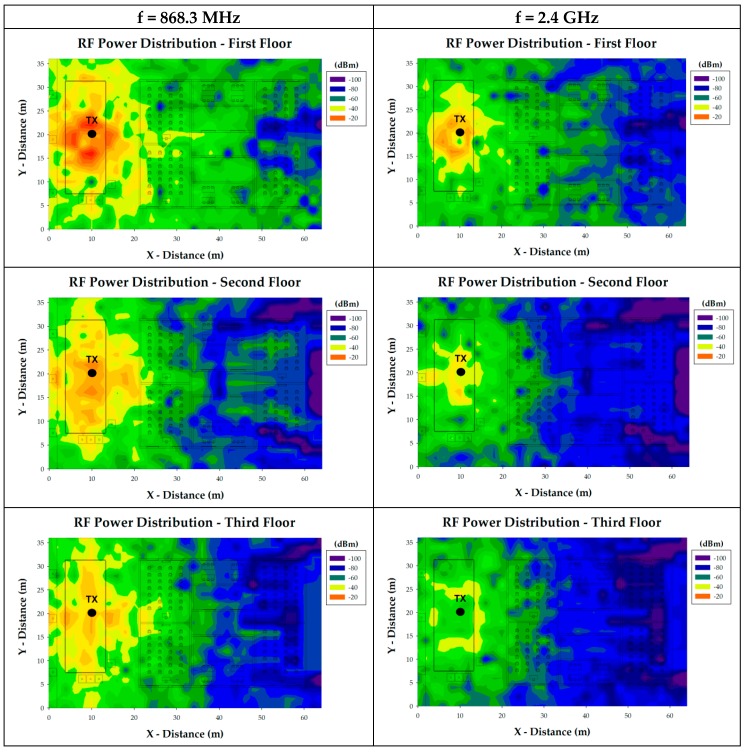
RF power distribution plane comparison between 868.3 MHz and 2.4 GHz frequency for a single wireless sensor placed on the orchard (TX).

One of the issues which were taken into account when wireless sensors were chosen, apart from the self-powered feature, was the operation frequency of the transceivers. As presented in the Introduction section, in the literature almost all WSNs deployed for Smart Agriculture use ISM 2.4 GHz band for the wireless communication. The EnOcean STM 330 devices (EnOcean GmbH, Oberhaching, Germany) used in this work operates at 868.3 MHz, which means that in same conditions (same transmitted power level and antenna), the coverage or reach of these nodes is higher than those operating at higher frequencies. This could be a key aspect in a star topology WSN since it is mandatory to have direct communication between the gateway and each of the wireless sensors of the network. In this way, further simulations have been performed in order to compare the performance between EnOcean STM 330 modules and a common ZigBee module operating at 2.4 GHz. The used simulation parameters are summarized in Table 1. The obtain simulation results for a wireless sensor placed in the centre of the orchard are shown in Figure 7. The RF power distribution planes at the height of the three floors of the school building are presented. As expected, 868.3 MHz shows better performance in terms of RF power level, both outside and inside the school building.

**Figure 8:**
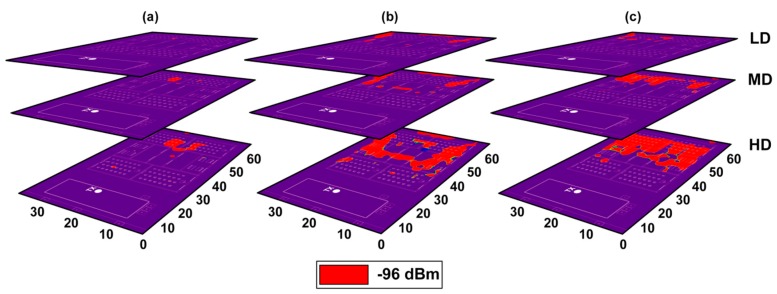
Fulfilment of the sensitivity for the central node potential placement, where sensitivity threshold is −96 dBm. (**a**) First floor; (**b**) Second floor; (**c**) Third floor.

This kind of results give a valuable information for a correct deployment of the proposed WSN, as the central node of the network will be deployed within the building. Since the sensitivity of the EnOcean USB 300 central node is −96 dBm, sensitivity fulfilment planes can be obtained based on the RF power distribution planes. In this case, at 868.3 MHz, the sensitivity threshold is never exceeded inside the building. This means that there are not restricted areas where the central node can be placed inside the building. But there is another important issue that has not been taken into account yet: the presence of human beings. It is well known that the presence of human body affects significantly the radio propagation due to its absorption properties, which creates the also well-known shadowing effect [42]. Depending on how many human beings are within a scenario (i.e., human body density), propagation losses vary greatly in indoor environments [43]. Based on this loss variability, in Figure 8 sensitivity fulfilment planes for approximated losses for different human body densities are presented. Three human body densities have been defined: LD (Low Density), MD (Medium Density) and HD (High Density), with their corresponding propagation losses: 10 dB, 20 dB and 30 dB respectively. The areas/zones that do not comply with the sensitivity requirements are highlighted in red, whereas the blue zone indicates that the EnOcean gateway could be deployed as the received power level is higher than the sensitivity threshold. Obtaining the sensitivity fulfilment planes for each of the deployed wireless sensors on the orchard, an optimum location for the gateway can be estimated. If the location of the gateway is restricted to specific classes or laboratories (which is usually the case), it can be assessed whether they are adequate places for the deployment or not. In this work, after obtaining all the sensitivity fulfilment planes, a laboratory and a teachers’ room in the second floor have been selected as the best choices to deploy the gateway of the WSN. It is worth noting that the first-floor areas beside the orchard were also a good option but they were discarded since they were common classrooms and were defined as inappropriate. 

**Figure 9:**
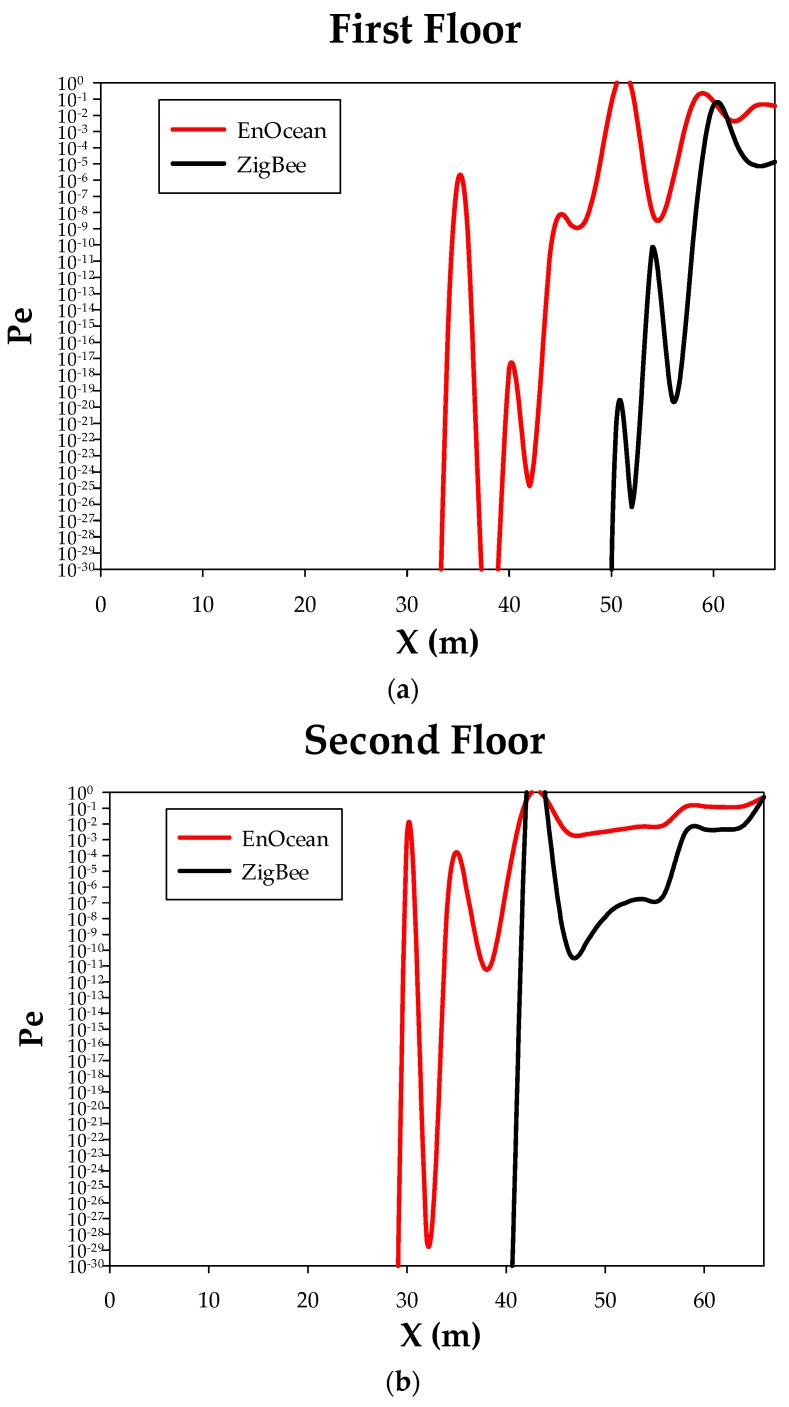

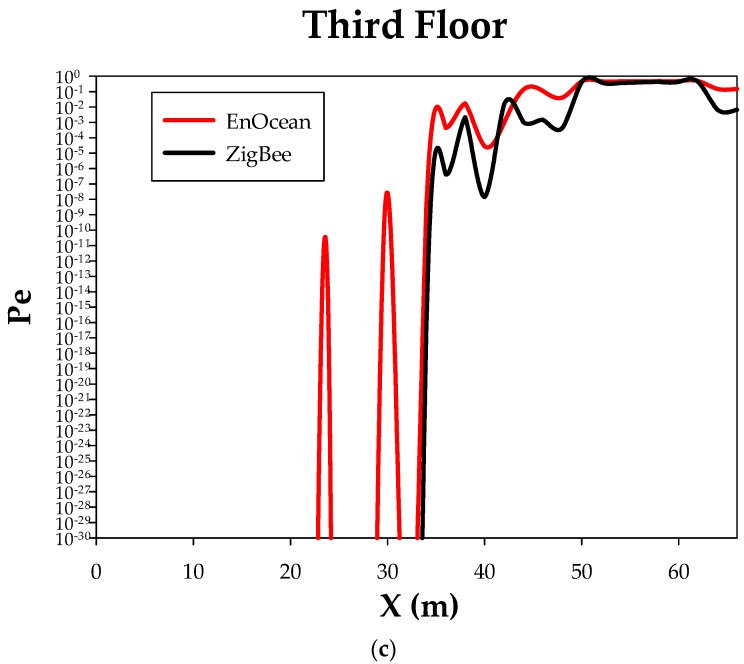
Performance comparison between EnOcean and ZigBee in terms of BER for the linear path depicted in Figure 5. (**a**) First floor; (**b**) Second floor; (**c**) Third floor.

For an in-depth performance analysis, BER (Bit Error Rate) values can be calculated for both EnOcean and ZigBee options. Figure 9 shows the obtained BER for the linear path depicted in Figure 5. For the estimations, a noise level of −70 dBm has been considered for both communication schemes. ZigBee shows a better performance in terms of BER when considering the same noise level, due to its bandwidth and modulation scheme (3 MHz, Q-PSK) in contrast to EnOcean’s 70 KHz bandwidth and ASK modulation. It is worth noting that the error probability is almost zero outdoor and it starts to be higher for locations within the building, which is expected. But in general, the obtained error probability is very low except in locations at the far end of the building, as it happened for the received power distributions (see Figure 7).

## 4. Application Design and System Validation

**Figure 10:**
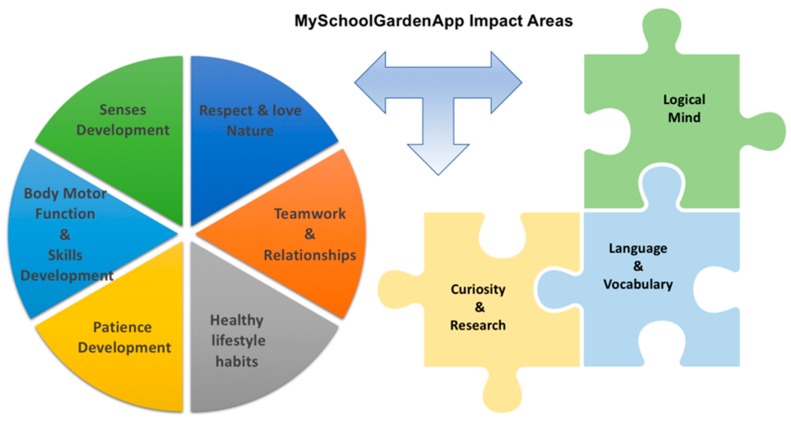
MySchoolGardenApp Impact Areas.

This section presents the developed educational application based on the EnOcean WSN deployed on school gardens. School gardens are becoming increasingly popular in urban districts as they are a very useful educational resource for urban schools. They boost students’ interest in learning specific botanical concepts by allowing them to engage in agricultural practices on a small scale and they help students become responsible caretakers by teaching them the responsibilities and impacts of land cultivation. Besides, the students start using and learning technological concepts such as sensors, wireless communications and clouds. But the benefits of school gardens as complementary educational tools do not stop there. The application presented in this work, called MySchoolGardenApp, covers a large number of educational impact areas in addition to those previously presented. For instance, it increases students’ interest in several topics including the following: the value of the natural environment and its importance in human life, the history of different crops, mathematics for the calculus of surfaces, weights collected, number of fruits and, in higher courses, the use and interpretation of graphs on time and the Internet search on different cultivation methods. Figure 10 describes the areas MySchoolGardenApp has the largest impact on.

**Figure 11:**
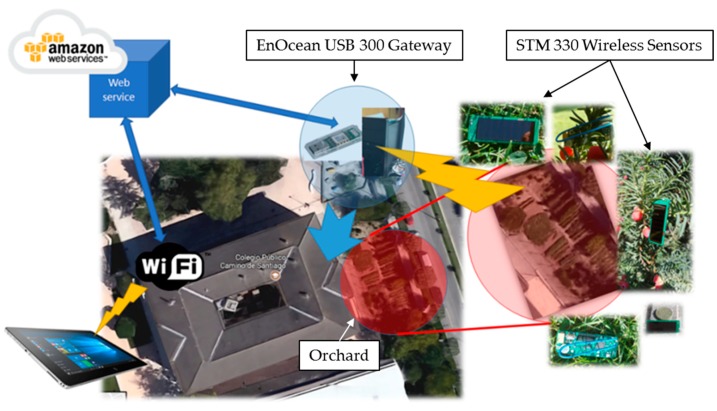
Scheme of the system architecture.

A simple system architecture is required to support the application (see Figure 11). EnOcean’s STM 330 nodes gather the information and transfer it wirelessly (868.3 MHz) to a gateway node located in the building. This USB-based gateway is connected to a PC, which is in charge of the collection and management of data and its transmission to the cloud. This channel is also used to provide control commands to the sensor nodes when needed. The used PC is an Ubuntu 16.4 Linux computer that uses a RESTful API to transfer data to the cloud (Amazon) by means of a web service. Basically, the periodically collected information is transferred to the cloud. In addition, meteorological information obtained from a web service run by the Government of Navarre (http://meteo.navarra.es) is used to complement the locally collected data (temperature). Through this efficient data acquisition process, all the information the application needs (including the meteorological information and the harvested data) is statistically merged and processed using Amazon’s RDS (Relational Database Service) and RStudio Server. Finally, access to the generated reports, statistical data analysis tools and all user process capabilities and features (restricted by the user profile) are available through mobile devices (smartphones and tablets), electronic boards and computers using the Wi-Fi network of the building.

**Figure 12:**
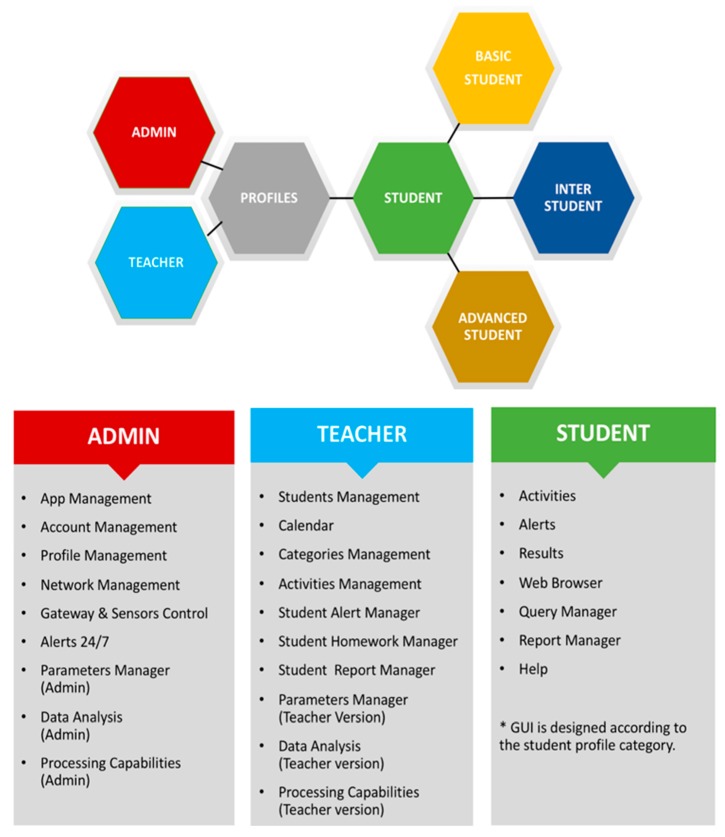
MySchoolGardenApp Profiles and General Tasks.

From a software level view, MySchoolGardenApp has different Graphical User Interfaces (GUIs) to accommodate different user types. The different GUIs allow the system to restrict data access and function usage based on a given user’s user type. An Administrator profile is available to control the performance of the application by monitoring features and accounts and making adjustments if necessary. The Administrator is also responsible for the management and maintenance of the hardware infrastructure sensor network, the gateway, communications and the software licenses required for data processing (Amazon RDS and RStudio Server). At the operational level, Teacher and Student profiles are available. Teachers are able to exploit the application’s features to design activities, set and manage schedules and alerts and analyse data to assess students’ performance. Students, similarly, can benefit greatly from the application by using its interactive learning tools. The students are further broken into the following three categories based on their grade level: basic, intermediate and advanced. This division allows the system to be a much more customizable tool for teachers, who can then assign content suitable for the educational needs of their students based on their students’ levels of understanding. User profiles and their general tasks are presented in Figure 12.

**Figure 13:**
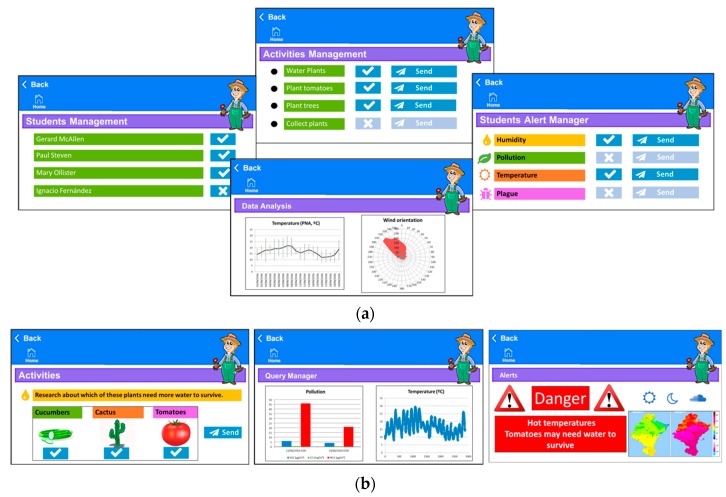
MySchoolGardenApp GUI. (**a**) Teacher profile GUI; (**b**) Student profile GUI.

MySchoolGardenApp is designed to be as simple and intuitive as possible. Teachers can assert their charge of the maintenance of the urban school garden by using the application’s activities management system to assign tasks to students. They can interact in real-time with their students, creating, sending or evaluating activities, alerts or homework through the application’s interface that they can access using their own personal portable devices. Some panels from the applications’ Teacher GUIs are shown in Figure 13a. Students can query information on the app through their own devices. The content made available to a given student by the app depends on the age and knowledge level of that student. For example, the system provides easy-to-understand pictograms appropriate for younger students (Basic Student Profile) whereas it provides comparative tables of the evolution of multiple variables measured by sensors, advanced graphs and even regressions and trends for older students (Advanced Student Profile). Figure 13b shows some panels from Student profiles’ GUIs including those for results queries, activities and alerts. The graphs presented are aimed at students who are already able to understand graphs of a certain complexity with their knowledge of statistics.

## 5. Conclusions

In this work, an environment to enhance School Garden observation for educational purposes in primary school has been presented. The system is formed by a set of autonomous wireless sensor nodes, which transmit information to a cloud capable platform. The selected nodes, the EnOcean STM 330 modules, present very interesting features compared to the typical ISM 2.4 GHz ZigBee nodes used in the literature in order to monitor agricultural tasks: They are self-powered by an incorporated small solar cell, the whole wireless node (sensors plus wireless transceiver) is very small and the operation frequency of 868.3 MHz gives a longer range of direct communication.

Pre-deployment deterministic wireless channel analysis results have been obtained by means of 3D Ray Launching in-house simulations in order to estimate coverage relations for the proposed wireless communication system. Operation is feasible for both operating frequencies, in which the communications between point to point links for the employed star network configuration are fulfilled in terms of receiver sensitivity thresholds. Moreover, the influence of persons within the school environment has also been taken into account, revealing that it is a key issue since the overall losses due to human body presence reduces significantly the effective coverage range of the system.

On the other hand, an ad-hoc application called MySchoolGardenApp has been implemented, in order to monitor and process the obtained observation data from the sensors located on a garden located within the school yard of a primary school. The app offers different GUIs and possibilities depending on the user’s profile (Administrator, Teacher or Student), which make the app flexible and simple to use. Initial testbed results have been obtained, showing the feasibility of the proposed system, which can provide multiple and adaptive results, tailored to the specific classroom needs.

It is important to note that feedback regarding the system operation and the MySchoolGardenApp is missing for the case when academic activities are being carried out, which will be determinant in order to validate the proposed solution and develop further work. Future developments of this work are the application of the presented system to higher educational levels, such as university courses, monitoring the crops used for agronomical engineering studies (which will have more difficulties due mainly to the big surfaces they occupy) and the adaptation of the application to such university course levels.

## Figures and Tables

**Table 1:** 3D Ray Launching simulation parameters.

Parameter	868.3 MHz	2.4 GHz
Output power level	8 dBm	8 dBm
Antenna type	Whip antenna	Whip Monopole
Antenna Gain (average)	0.2 dB	0.2 dB
Permitted reflections	6	6
Cuboid resolution	2 m × 2 m × 2 m	2 m × 2 m × 2 m
Launched Rays resolution	1°	1°
Data rate	125 kbps	250 kbps
